# Machine learning classifier for identification of damaging missense mutations exclusive to human mitochondrial DNA-encoded polypeptides

**DOI:** 10.1186/s12859-017-1562-7

**Published:** 2017-03-07

**Authors:** Antonio Martín-Navarro, Andrés Gaudioso-Simón, Jorge Álvarez-Jarreta, Julio Montoya, Elvira Mayordomo, Eduardo Ruiz-Pesini

**Affiliations:** 1 Departamento de Bioquímica, Biología Molecular y Celular, Universidad de Zaragoza, C/ Miguel Servet 177, Zaragoza, 50013 Spain; 2 Departamento de Informática e Ingeniería de Sistemas, Universidad de Zaragoza, C/ María de Luna 1, Zaragoza, 50018 Spain; 3 Instituto de Investigación en Ingeniería de Aragón (I3A), Universidad de Zaragoza, Zaragoza, Spain; 4 Instituto de Investigación Sanitaria de Aragón (IISA), Universidad de Zaragoza, Zaragoza, Spain; 5 Centro de Investigaciones Biomédicas en Red de Enfermedades Raras (CIBERER), Universidad de Zaragoza, Zaragoza, Spain; 6 Fundación ARAID, Universidad de Zaragoza, Zaragoza, Spain

**Keywords:** Mitochondrial DNA, Missense mutation, Pathogenicity, Classifier, SVM, Protein multiple sequence alignment

## Abstract

**Background:**

Several methods have been developed to predict the pathogenicity of missense mutations but none has been specifically designed for classification of variants in mtDNA-encoded polypeptides. Moreover, there is not available curated dataset of neutral and damaging mtDNA missense variants to test the accuracy of predictors. Because mtDNA sequencing of patients suffering mitochondrial diseases is revealing many missense mutations, it is needed to prioritize candidate substitutions for further confirmation. Predictors can be useful as screening tools but their performance must be improved.

**Results:**

We have developed a SVM classifier (Mitoclass.1) specific for mtDNA missense variants. Training and validation of the model was executed with 2,835 mtDNA damaging and neutral amino acid substitutions, previously curated by a set of rigorous pathogenicity criteria with high specificity. Each instance is described by a set of three attributes based on evolutionary conservation in Eukaryota of wildtype and mutant amino acids as well as coevolution and a novel evolutionary analysis of specific substitutions belonging to the same domain of mitochondrial polypeptides. Our classifier has performed better than other web-available tested predictors.

We checked performance of three broadly used predictors with the total mutations of our curated dataset. PolyPhen-2 showed the best results for a screening proposal with a good sensitivity. Nevertheless, the number of false positive predictions was too high. Our method has an improved sensitivity and better specificity in relation to PolyPhen-2. We also publish predictions for the complete set of 24,201 possible missense variants in the 13 human mtDNA-encoded polypeptides.

**Conclusions:**

Mitoclass.1 allows a better selection of candidate damaging missense variants from mtDNA. A careful search of discriminatory attributes and a training step based on a curated dataset of amino acid substitutions belonging exclusively to human mtDNA genes allows an improved performance. Mitoclass.1 accuracy could be improved in the future when more mtDNA missense substitutions will be available for updating the attributes and retraining the model.

**Electronic supplementary material:**

The online version of this article (doi:10.1186/s12859-017-1562-7) contains supplementary material, which is available to authorized users.

## Background

Mitochondrial DNA (mtDNA) encodes 13 key polypeptides of the oxidative phosphorylation (OXPHOS) system. Its close proximity to a major reactive oxygen species (ROS) source and its particular replication system favours a quick accumulation of mutations [1]. Since polypeptide-coding genes represent 70% of the human mtDNA, most of these mutations affect protein-coding genes. Many mutations in protein-coding genes are missense point mutations (non-synonymous) that provoke an amino acid substitution. Some of these mutations will produce very severe disorders, but many others will have no important phenotypic effects. It is not an easy task to differentiate the former from the latter, and several criteria have been proposed to achieve this goal [1–3].

The functional characterization of a mtDNA missense mutation is an irreplaceable way to determine its phenotypic effect and potential pathogenicity, but this process is not always possible and is expensive and time consuming. Moreover, because mitochondrial disorders can also be due to nuclear DNA (nDNA) mutations [4], the finding of ways to prioritize which mtDNA mutations should be subjected to functional analysis is crucial. For this reason, computational predictors are useful, inexpensive and fast tools for checking novel missense mutations reported in patients with a possible mitochondrial disease and assist in the selection of mutations for subsequent functional assays.

Different prediction algorithms and web tools are available to classify missense mutations in neutral or damaging categories [5]. Unfortunately, the overall accuracy of these tools is low, and predictions generated by current computational tools may mislead researchers involved in downstream experimental and clinical studies [6–8]. Moreover, most of these predictors focus uniquely on the nuclear genome. As an example, Mutpred [8] and PolyPhen-2 [7] use Human Genome Mutation Database (HGMD) [9] and HumDiv dataset, respectively, to train the program. Provean [10] uses Humsavar dataset [11] to adjust cut off of damaging mutations. These datasets do not include, or only include a few, mtDNA missense variants and do not consider the special features of mtDNA-encoded polypeptides.

Our goal is to develop a classifier to select damaging missense candidates of the human mtDNA genome with improved performance. Our validation shows that our method Mitoclass.1 is a good alternative to other web-available tools.

## Methods

### Pathogenicity criteria for identification of damaging amino acid substitutions

The dataset of damaging and neutral mtDNA missense mutations was obtained from the Mitomap website (February 2015). However, many missense variants reported in the Mitomap section “mtDNA Mutations with Reports of Disease-Associations” meet only a small number of the established pathogenicity criteria for mtDNA mutations [1, 12] and their pathogenicity is, therefore, doubtful. To solve this problem, we have only considered as damaging missense mutations those substitutions that have been associated with a possible mitochondrial disease and meet, at least, one of these two pathogenicity criteria:

a) Functional confirmations. It has been previously reported that functional studies provide high quality evidence in support of pathogenicity [13]. Therefore, cellular, biochemical and molecular-genetic studies of mtDNA missense mutations, using cybrids or single fiber analysis, are important ways to determine their phenotypic effects and potential pathogenicity. With both techniques, different mtDNA genotypes are associated to a same nuclear genetic background and environment. Then, the differences between cybrids or between single muscle fibers, with different mutational load, would be due to the mtDNA genotype.

b) Rareness of the mutation. Rare diseases affect a limited number of individuals, defined as no more than one in 2,000 individuals in the European Union [14]. Mitochondrial disorders are rare diseases and are present clinically in at least one in 10,000 adults [15]. Thus, we have also considered damaging changes those mutations present in more than one pedigree with patients suffering from mitochondriopathy but absent in the control population, or at a very low population frequency (≤0.1‰), then suggesting an association with the pathology. Moreover, because negative selection tends to remove deleterious mutations along the evolution, these mutations must be absent at internal branches of a phylogenetic tree.

After the application of these criteria to missense variants reported in the Mitomap section “mtDNA Mutations with Reports of Disease-Associations”, we generated the mtDNA missense variants.1 (mdmv.1) dataset including 57 damaging and 2,778 neutral variants (Additional file 1: Table S1). These neutral variants include those mutations from the Mitomap section “mtDNA Mutations with Reports of Disease-Associations” that do not fulfill the above criteria (85 variants), and those from Mitomap section “mtDNA Variants”.

### Identification of positions belonging to the same domain for the 13 human mtDNA-encoded polypeptides

All the human mtDNA-encoded polypeptides are integral membrane proteins with intermembrane (IM), transmembrane (TM) and matrix (M) domains. The biochemical environments of these three regions are different. Thus, same amino acid substitutions in different domains will not have the same functional effects [16]. Unfortunately, there are no crystal structures available for these human polypeptides. Therefore, domain characterization of these thirteen polypeptides is made using structural homology with ortholog proteins from other organisms, mainly bacterial species, for which the crystal structure has been published.

The p.MT-ATP8 polypeptide is a supernumerary polypeptide not present in prokaryote species. Moreover, there is no published p.MT-ATP8 crystal structure from other eukaryote species. In this case, we have used information contained in previous studies [17].

Domains are annotated with the help of Cn3D 4.3.1 visualization tool [18]. This program allows correlating sequence, alignment and structure information. In this way, we correlate crystal visualization for each ortholog protein with the alignment between it and its human mtDNA-encoded polypeptide counterpart. With this methodology, we identify the first and last positions of each transmembrane helix. Using Jmol visualization of the complete respiratory complex crystals published in other species, we can determine the domain of the N and C terminus of the polypeptides (Additional file 2: Table S2 and Additional file 3: Table S3).

### Discriminatory features

#### Feature 1. Conservation index + cumulative Mutual Information in Eukaryota

This feature is the sum of two scores: Conservation index + cumulative Mutual Information. For conservation index (CI) analysis, we used multiple sequence alignments of orthologs.

The revised Cambridge reference sequence (rCRS, NC_012920.1) was used to define the reference amino acid sequence of each gene. We used Bioperl Eutilities [19] to retrieve from GenBank [20] all mtDNA-encoded polypeptides for organisms of the RefSeq database (around 5,000 species on February 2015). Multiple sequence alignment of orthologs was made with MAFFT v.7.147b (−−auto option) [21].

Each aligned fasta file of ortholog sequences was used as the input argument of a Perl/Awk script to calculate CI, which was defined as the relative frequency of the amino acid present in the RefSeq human polypeptide. Values are represented as a percentage.

For coevolution analysis, we used MISTIC (mutual information server to infer coevolution), a web server for a complete analysis of Mutual Information networks in protein families [22]. Mutual Information (MI) from information theory can be used to estimate the extent of the coevolutionary relationship between two positions in a protein family. The cumulative Mutual Information score (cMI) defines to what degree a given amino acid takes part in a mutual information network and is provided by the program. Because cMI values differ from one polypeptide to another, we have considered a relative cMI with a normalized scale from 0 to 100% defining as baselines the minimum and maximum cMI values for each polypeptide.

#### Feature 2. Conservation of the mutant amino acid for each single position in the polypeptides

Relative frequency of each mutant amino acid for every amino acid position of the polypeptides was calculated. Information was obtained from the multiple alignment files. Each aligned fasta file of ortholog sequences was used as the input argument of a Perl/Awk script to calculate absolute and relative frequency of each mutant amino acid, including gaps, for all positions.

#### Feature 3. Relative frequency for each variant into a particular domain

Given that the thirteen polypeptides encoded by the human mtDNA are integral membrane proteins with three distinct domains (IM, TM and M), we generated a matrix of conservation of each amino acid for the three domains with the multiple alignments of the thirteen polypeptides using relative frequencies of each mutant amino acid obtained for feature 2. Next, all positions belonging to the same domain were grouped to generate a table of relative frequencies of variants for each possible amino acid. Finally, a new table was generated to exclude gaps frequency and conservation of each amino acid (diagonals of the table) from calculation of the final score for relative frequency of the variants (Additional file 4: Table S4).

#### Feature evaluation

We evaluated attributes with Weka (Select Attributes). We selected the complete training database and applied the attribute evaluator CfsSubsetEval together with the search method Best First.

### Machine learning method

The complete curated dataset of 2,835 missense variants (mdmv.1 dataset) was split randomly into two: a training dataset (60% damaging / 60% neutral variants) and a validation dataset (40% damaging / 40% neutral variants) (Additional file 5: Table S5). We use a Support Vector Machine (SVM) Classifier [23]. The open-source data mining suite WEKA 3.7.7 [24] was selected for execution of the classification algorithms. Numerical values of features were normalized (that is, rescaled to the range of 0 to 1) before using the classifier. Parameters “C” and “gamma” were optimized by a grid search using 10-fold cross validation of training dataset. Finally, C = 200 and gamma = 0.01 were selected. Ten folds cross validation was also executed with the training dataset to check the robustness of the method and prevent the possibility of overfitting. Because of the imbalanced nature of training dataset (damaging and neutral mutations are not represented equally) we used SMOTE [25] (parameters: nearestsneightbors = 5; randomseed = 1) to oversample the minority class (damaging mutations) in order to have a similar number of mutations from both classes (Additional file 6: Table S6). SMOTE works by creating synthetic samples from the minor class instead of creating copies. The algorithm selects two or more similar instances (using a distance measure) and perturbing an instance one attribute at a time by a random amount within the difference to the neighboring instances.

The minority class must be properly oversampled (Additional file 7: Figure S1).

We have also generated a repository in Github with information about the data/scripts used: https://github.com/tonomartin2/MITOCLASS.1/.

### Evaluation of the predictor

To evaluate the generalization of the SVM model, a blind test was carried out with the validation dataset, which was not involved in the training process. Sensitivity and specificity were calculated.

Our validation dataset is a subgroup of mdmv.1, the self-curated dataset containing only mtDNA missense variants. Several benchmark datasets exist for nuclear mutations classifiers. These benchmark datasets cannot be used in our work for validation/training purpose because we are interested in a screening classifier specific for mtDNA substitutions not including nuclear variants. Moreover, discriminatory features of our classifier are not possible to be calculated for nuclear substitutions.

Predictive results of SVM on the validation dataset were compared with results of other classifiers. The comparison was made using the default parameters offered by Weka for other classifiers. We selected Random forest, IBK, SMO and Naive Bayes Multinomial.

### Statistical analysis

Mann-Whitney-Wilcoxon test has been run with R language to decide whether the population distributions (damaging and neutral) are identical without assuming them to follow the normal distribution (*P* ≤ 0.05 as significance level).

## Results and discussion

### Selected features for the discrimination of neutral and damaging mutations

#### Feature 1: Conservation index of the human wild-type amino acid + cumulative Mutual Information in Eukaryota

Conservation index (CI), defined as the frequency of the reference human amino acid at a particular position in Eukaryota species, is a commonly used attribute to determine pathogenicity of amino acid substitutions in mtDNA-encoded polypeptides [1, 2]. Many recent methods have used conservation along with other parameters to predict functional importance but it has been found that conservation is the single most powerful attribute [26, 27].

To perform this analysis, it is required an alignment of ortholog polypeptide sequences and a reliable metric for quantifying residue conservation. Many scores have been proposed, but none has emerged as a generally accepted standard [28]. Moreover, it has been demonstrated that predictions of impact on protein structure and function for missense mutations depend on which values are chosen for alignment parameters [29]. Other predictors use Basic Local Alignment Search Tool (BLAST) and the human reference polypeptide as the query sequence to find a list of sequence-similar proteins that are potential homologs. Therefore, paralogs and nuclear pseudogenes of mtDNA-encoded polypeptides (NUMTs) could be also retrieved because of sequence similarity. Moreover, low identity orthologs from evolutionary distant species could not be recovered depending on the BLAST parameters considered. To avoid any selection bias, we have decided to retrieve all the ortholog sequences from organisms of the RefSeq database: 4,668 for p.MT-ATP8 and 5,177 for p.MT-ND6 are the lowest and highest numbers of analyzed species for the 13 mtDNA-encoded polypeptides. These differences are mainly due to the fact that the 13 mtDNA-encoded human polypeptides are not mtDNA-encoded in all organisms. Moreover, there are a minority number of sequences that have not been published in GenBank database with the same “protein name” tag and could not been retrieved by our searching protocol.

The CI analysis for a particular position in a mtDNA-encoded polypeptide is an inexpensive and quick approach to obtain information on its functional significance. The underlying idea is that an important position will be highly conserved throughout the evolution because mutations in this location will be removed by negative selection. As an example, histidines 83, 97, 182 and 196 from p.MT-CYB and 61, 376 and 378 from p.MT-CO1 that bind the heme groups required for the electron transfer reactions in the respiratory chain show CI values equal or higher than 99.8% in our panel of organisms (Additional file 8: Table S7). Thus, the mean CI of amino acids affected by damaging variants [79.0% ± 29.0 (57)] is significantly higher (*P* = 6.64e^−15^) than that of neutral variants [41.4% ± 34.2 (2778)] (Additional file 1: Table S1).

However, a certain percentage (14.3%) of neutral variants of mdmv.1 dataset show really high (≥90%) CIs. In some cases, these results might mirror sequencing errors or misclassification of the variants. mtDNA sequences are very frequently obtained in population studies from many individuals and a special attention to the quality of the sequence is, sometimes, not paid [30]. Moreover, the incomplete penetrance of some mtDNA missense variants can make difficult to classify them correctly. As an example, the m.11778G > A transition causes a p.MT-ND4: Arg340His substitution and provoke Leber hereditary optic neuropathy (LHON) [31]. The CI of this p.MT-ND4: Arg340 is 99.2%. However, many individuals from a LHON pedigree harboring this mutation do not develop the disease because other factors are required to express the phenotype [32]. Then, if a pathologic mutation is found in a healthy individual from a population study and information on his/her family is not recovered, this damaging mutation might be wrongly considered neutral.

A high CI for a particular polypeptide position gives an idea about its functional importance. However, a low CI does not directly imply lack of functional importance. In fact, a small number (12.3%) of damaging mutations show really low (≤25%) CIs. Thus, it is possible that an amino acid substitution (A to B) in a polypeptide position X is compensated, along the evolution, by a change (C to D) in other position Y of the same or other polypeptide [33]. This fact would allow the fixation of a new amino acid (B) in that particular position X and a lower CI for A, although that position was functionally important. To check this possibility, we estimated the cumulative Mutual Information (cMI) score, which is representative of the covariation along the evolution. In fact, the 7 damaging mutations with CIs ≤ 25% have cMIs (57.5% ± 11.0) significantly higher (*P* = 0.0088) than those of 1,278 neutral mutations with CIs ≤ 25% (44.1% ± 21.1) (Additional file 1: Table S1). As an example, the m.10663 T > C transition causes a p.MT-ND4L: Val65Ala substitution and provokes LHON [34]. The CI of this p.MT-ND4L:V65 is 24.7% but its cMI is 78.3%. Therefore, the low CI of some functionally important amino acids is probably due to environmental or genetic coevolution [35].

In order to consider amino acid conservation and coevolution with other amino acids together, we created a feature including both of them (Feature 1 = CI + cMI). There are significant differences (*P* = 5.37e^−10^) in this feature between damaging [107.9% ± 22.6 (57)] and neutral [80.2% ± 33.8 (2778)] variants (Additional file 1: Table S1).

#### Feature 2: Conservation index of the human mutant amino acid

A similar consideration about evolutionary conservation and functional importance can be applied for the mutant amino acid. Thus, some amino acids would be very deleterious in particular polypeptide positions and natural selection would tend to remove them. Therefore, its relative frequency would be very low. In this sense, the mean relative frequency of the new amino acid is significantly lower (*P* = 1.893e^−14^) in the damaging [1.4% ± 5.1 (57)] than in the neutral [8.2% ± 14.2 (2778)] mutations (Additional file 1: Table S1). Interestingly, 12 out of 57 (21.1%) damaging mutations have substituted an amino acid by another one not found in our multi-species (close to 5,000 sequences) alignment [in 6 cases (50%), the new amino acid was proline] but this has only occurred in 137 out of 2778 (4.9%) neutral mutations [in 14 cases (10.2%), the new amino acid was proline].

#### Feature 3: Relative frequency of specific amino acid substitutions into a particular domain

This feature tries to quantify the importance of a specific amino acid substitution (for example, alanines by threonines). In general, mutant amino acids chemically similar to the substituted ones will be less affected by natural selection than those very different. However, amino acids have many different physicochemical and biochemical properties. Thus, an amino acid can share some properties with certain amino acids and others with different amino acids [36]. Therefore, it is not an easy task to select the best discriminative properties. We can take advantage of evolution. Thus, if a specific substitution is rarely observed, this might indicate that key properties of the new amino acid are very different from those of the original one and natural selection tent to remove it. However, not all amino acid substitutions are equally plausible. Thus, some particular amino acid changes require two mutations in the same codon, a very improbable event. Other amino acid replacements require DNA transversions but, in animal mtDNA, transitions are much more common [37]. This fact would introduce a bias in the frequency of particular substitutions. In any case, this observation is independent of the phenotypic effect of a particular amino acid exchange. Moreover, we have observed that there are significant differences (*P* = 0.0003) among relative frequencies of specific substitutions associated [9.4 ± 8.8 (57)] or not [12.7 ± 8.5 (2778)] to pathologic mutations (Additional file 1: Table S1).

The human mtDNA-encoded polypeptides are integral membrane proteins with three distinct domains (IM, TM and M). These environments are very different and, therefore, the same amino acid substitution can present different effect depending on the affected domain, as it has been previously documented [38]. Then, we have previously defined the mtDNA-encoded polypeptide domains (Additional file 2: Table S2). Next, we determined the relative frequency of each amino acid in every domain (Table 1). L and T have frequencies > 8% in the three domains. A and I in the TM domain; and P in the IM and M domains have also frequencies > 8%. In general, and as expected, the TM domain is enriched (2/3) and impoverished (1/3) in hydrophobic and hydrophilic amino acids, respectively. C, K and R in the IM; C, D, E, K, Q, and R in the TM; and C in the M domains have frequencies < 2%. Remarking the inter-domain differences, the CIs for several amino acids are very dissimilar between domains. Thus, inter-domain differences in the E, H, K, M, W and Y CIs are > 15% (Additional file 4: Table S4). For example, the CIs for Y in the IM and M domains are 67.1 and 43.2%, respectively. These observations suggest that a Y substitution will probably have a more important phenotypic effect in the IM than in the M domain.

**Table 1:** Amino acid (AA) relative frequency (%) and conservation index (CI)

	IM					TM					M				
AA	N	%	CI	tM	D	N	%	CI	tM	D	N	%	CI	tM	D
A	37	5.1	50.4	37	0	192	8.3	57.4	177	7	26	3.5	44.6	27	1
C	3	0.4	67.4	1	0	14	0.6	59.2	8	0	5	0.7	64.5	4	0
D	28	3.8	73.2	25	0	21	0.9	69.8	9	2	17	2.3	72.4	16	0
E	18	2.5	57.5	7	0	42	1.8	78.4	23	2	28	3.8	71.0	15	2
F	30	4.1	60.7	22	0	154	6.7	69.5	104	1	32	4.3	58.3	18	1
G	46	6.3	73.9	25	0	128	5.5	80.8	60	4	38	5.1	77.0	15	0
H	18	2.5	64.6	12	0	51	2.2	74.0	22	1	28	3.8	55.9	26	0
I	45	6.2	44.4	44	0	234	10.1	46.0	318	0	40	5.4	35.5	55	0
K	11	1.5	58.2	1	0	39	1.7	66.2	9	0	45	6.0	45.3	17	0
L	98	13.4	50.1	44	1	457	19.7	60.4	205	12	89	11.9	49.0	46	1
M	31	4.3	52.5	20	2	140	6.1	38.9	134	3	37	5.0	34.1	36	0
N	49	6.7	48.4	49	0	65	2.8	47.7	59	0	50	6.7	44.0	70	0
P	61	8.4	70.9	31	0	90	3.9	65.1	41	2	68	9.1	65.2	41	0
Q	24	3.3	55.9	8	0	35	1.5	63.2	11	1	31	4.2	54.1	20	0
R	10	1.4	86.6	2	0	33	1.4	80.5	14	1	20	2.7	82.6	13	3
S	58	8.0	39.6	55	0	159	6.9	53.1	117	2	57	7.6	42.2	61	2
T	82	11.2	36.3	73	0	207	8.9	40.6	201	0	62	8.3	33.4	79	0
V	33	4.5	49.7	32	0	114	4.9	50.7	147	3	20	2.7	42.0	18	0
W	20	2.7	89.0	1	0	64	2.8	79.9	19	1	20	2.7	71.5	7	0
Y	27	3.7	67.1	17	1	75	3.2	64.9	36	0	33	4.4	43.2	31	1
	729			506	4	2314			1714	42	746			615	11

IM, TM, M, N, tM and D code for intermembrane, transmembrane, and matrix domains, total positions with this amino acid, number of total and damaging mutations, respectively

The number of mutations per amino acid is similar between domains (Table 1). This number is 0.7 in TM and IM domains (1714/2314 and 506/729) and 0.8 in M domain (615/746). There are no differences in the frequencies of pathologic mutations. However, TM and IM domains show the highest (42 out of 1714, 2.5%) and lowest (4 out of 506, 0.8%) frequencies of pathologic mutations, respectively. Pathologic mutations in IM, TM and M domains affect 3 (L, M and Y), 14 (A, D, E, F, G, H, L, M, P, Q, R, S, V and W) and 7 (A, E, F, L, R, S and Y) amino acids, respectively (Table 1). Pathologic mutations affecting L are found in all the domains probably because this is the most frequent amino acid in each domain. Mutations in five amino acids have not been found associated to mitochondriopathies (C, I, K, N and T). Pathologic mutations affecting C are not found in any domain probably because this is the least frequent amino acid in each domain. Curiously, T is between the three most frequent amino acids in each domain and it is one of the three amino acids that more mutations have suffered, but no pathologic mutation has been associated to this amino acid. These results suggest that functional effect of substitutions on this T amino acid is not very important. On the contrary, R is one of the least frequent amino acids in the TM and M domains, but it shows pathologic mutations in both of them, thus remarking their functional importance.

There are significant differences (*P* = 0.0009) among relative frequencies of specific substitutions associated [9.3 ± 8.7 (42)] or not [13.3 ± 9.4 (1672)] to pathologic mutations in the TM domain. This does not occur in IM and M domains. In the TM domain, the substitution of a very frequent L by a very rare R is not frequent (0.3%) (Additional file 4: Table S4). This might be due to the fact that L to R change requires a transversion, although the substitution of a frequent M to a very frequent I is also very frequent (17.0%) and is due to a transversion. However, all the three domains contain substitutions of very frequent L to very frequent P. This change is produced by a transition, but the relative frequency of this particular substitution is very low (≤3.8%). These L to P mutations are associated many times with pathological phenotypes. L promotes generation of alpha-helix but P provokes the loss of hydrogen bonds in the peptide bonds, destabilizes the secondary structure and introduces a kink in this structure [39, 40]. From 10 described L-P damaging mutations, 8 are present in TM domain, in which most the mtDNA-encoded polypeptides acquire an alpha-helix conformation (Additional file 1: Table S1).

These three features were evaluated by the attribute selection tool of Weka and were considered useful and not redundant for training the classifier (Additional file 9: Figure S2).

### Assessment of the classifier

Firstly, predictive results of SVM on the validation dataset were compared with results of other classifiers. We did not get as good results as with the SVM (4.3% false negative predictions). We selected Random forest, IBK, SMO and Naive Bayes Multinomial with 39.1, 43.5, 87.0 and 17.4% of false negative predictions respectively.

Next, we have compared our classifier with other predictors, such as PolyPhen-2 (with HumDiv classifier model and both “probably damaging” and “possibly damaging” predictions considered as damaging), Provean (default settings) and with the results on mtDNA mutations previously reported using Mutpred (score cut-off 0.75) [41] (Table 2). These predictors are very popular and support batch submission, making them viable for analysis of a big set of mutations. First, we carry out a full analysis of these three predictors with mdmv.1 dataset. This analysis shows that PolyPhen-2 is the predictor with the best sensitivity (94.7%) and only 3 false negative predictions. Provean also has a high sensitivity (87.7%) and 7 false negatives. However, the sensitivity of Mutpred (cut-off ≥ 0.75) is very low (57.9%). Thus, 24 out of 57 pathologic mutations are not included in the damaging group by this predictor (Additional file 1: Table S1). Therefore, we ruled out Mutpred (with 0.75 cut-off) for the screening of mtDNA missense variants because it would remove too many potential damaging mtDNA mutations.

**Table 2:** Comparison between predictors with validation dataset of 1,100 mutations (23 damaging + 1,077 neutral)

	MITOCLASS.1	POLYPHEN-2	PROVEAN	MUTPRED
Sensitivity	95.7	91.3/94.7	91.3/87.7	60.9/57.9
Specificity	58.7	47.7/46.9	60.4/59.2	85.6/87.3
TP	22	21/54	21/50	14/33
TN	623	514/1303	650/1646	922/2426
FP	454	563/1475	427/1132	155/352
FN	1	2/3	2/7	9/24

For PolyPhen-2, Provean and Mutpred, the complete mdmv.1 dataset has also been analyzed (numbers after the slash). PolyPhen-2 is unable to predict the phenotype of 10 missense mutations because much of the initial and final sequence of p.MT-ND5 is non-aligneable due to large stretches of repeats and/or high compositional biases as commented by authors. For the sake of comparison, we consider these unknown predictions as neutral variants. TP, TN, FP, FN refers to true positives, true negatives, false positives and false negatives respectively. Sensitivity is estimated as [TP/(TP + FN)], specificity as [TN/(TN + FP)]

We cannot use the complete mdmv.1 dataset to evaluate our predictor because it includes mutations selected for training, so we use validation dataset to compare the performance of the four predictors (Additional file 10: Table 8). In this case, Provean and PolyPhen-2 show identical results in sensitivity (91.3%). Mitoclass.1 achieves a better sensitivity (95.7%) on the validation dataset. In addition, our classifier generates results for 100% of the analyzed variants. On the contrary, PolyPhen-2 does not generate predictions for ten mutations of p.MT-ND5 with result “unknown” for validation dataset and for 25 variants with result “unknown” when checking complete mdmv.1 dataset. A Venn diagram shows predictive results for the 23 variants confirmed as pathological present in the validation dataset. It can be observed that 13 (56.5%) have been classified as pathological by the four predictors (Additional file 11: Figure S3).

Analyzing false negative predictions of validation dataset, we observe that both Provean and PolyPhen-2 classified as neutral a mutation corresponding to the transition m.10158 T > C (p.S34P at p.MT-ND3). This mutation shows an inverse relationship in osteosarcoma 143B cybrids between the mutation load and the complex I activity [42]. Moreover, it has been reported in several pathologic pedigrees [42–45] and its pathogenicity confirmed [13]. The m.3700G > A transition (p.A132T at p.MT-ND1) is classified as neutral only by PolyPhen-2. This mutation is classified as a rare primary damaging mutation for LHON [46]. Moreover, Provean does not classify correctly as pathological the transversion m.4171C > A (p.L289M at p.MT-ND1), a primary LHON causative mutation [47]. These three mutations affect positions with low conservation in Eukaryota (Additional file 8: Table 7). Nevertheless, our classifier achieves a correct prediction for all of them probably because we do not use conservation of a single position as a discriminatory feature. By using sum of conservation and coevolution as an attribute (feature 1), we allow that little conserved positions but with significant signs of coevolution could be predicted as damaging. The only damaging mutation of validation dataset that our classifier does not predict as pathological is p.V65A at p.MT-ND4L. The reason is the very high relative frequency in eukaryotes (feature 2 = 35%) (Table 3).

**Table 3:** Analysis of features for false negative (FN) predictions of Provean, PolyPhen-2 and Mitoclass.1 in validation dataset

AA substitution (polypeptide)	FN	CI	F1	F2	F3
p.A132T (p.MT-ND1)	PolyPhen-2	72.82	123.98	0.30	12.59
p.L289M (p.MT-ND1)	Provean	29.72	78.64	1.99	16.07
p.S34P (p.MT-ND3)	PolyPhen-2 and Provean	10.15	60.53	0.34	5.23
p.V65A (p.MT-ND4L)	Mitoclass.1	24.72	103	35.06	6.78

CI refers to conservation index for each position. F1, F2 and F3 refer to the numerical values of the three attributes considered for Mitoclass.1 classifier

### Amino acid substitutions with no clear evidences of pathogenicity

For 37 mutations of validation dataset that Mitomap (http://www.mitomap.org) listed as “mtDNA Mutations with Reports of Disease-Associations”, we do not find enough evidences to classify them as really damaging and are included as neutral mutations in mdmv.1 dataset. For this set of variants, we analyzed the predicted results of the tested methods. Specificity of Mitoclass.1, PolyPhen-2, Provean and Mutpred for this group of mutations is 37.8, 43.2, 45.9 and 70.0%, respectively, compared to specificity for complete validation dataset: 58.7, 47.7, 60.4 and 85.6% respectively. Thus, the number of false positive predictions for this group is greater than in complete validation dataset. It indicates that some of these variants could be really damaging and additional confirmatory analysis would be interesting.

Nine out of 37 were classified as neutral by all four tested predictors and 5 more were classified as neutral by our and two other predictors, 3 of them with population frequencies > 0.1% (a frequency ten times higher than our established cut-off to separate pathologic from neutral mutations). On the other side, Mitoclass.1 classified 23 of them as damaging mutations (Additional file 12: Table S9, Additional file 13: Figure S4) and 16 of them are extremely rare in human beings (≤1 in 30589 human sequences, < 0.03‰). Moreover, 14 of these 16 mutations are considered damaging by 3 or more tested predictors and 11 were heteroplasmic mutations, another feature frequently considered as a pathogenicity criterium (Table 4).

**Table 4:** Feature values for rare missense mutations without clear evidences of pathogenicity classified as damaging mutations by Mitoclass.1

rCRS Mut	AA subs/PP/Dom	F1	F2	F3	Freq	Ho/He	DamPre
m.4633C > G	p.A55G/p.MT-ND2/TM	127.5	2.33	12.66	0	Ho	4
m.4648 T > C	p.F60S/p.MT-ND2/TM	113.7	0.02	4.22	0	Ho	4
m.5244G > A	p.G259S/p.MT-ND2/TM	138.9	1.01	23.65	0	He	4
m.6742 T > C	p.I280T/p.MT-CO1/TM	99.7	0.02	9.22	0	He	3
m.8528 T > C	p.W55R/p.MT-ATP8/M	102.6	0.04	14.99	0	He	4
m.8795A > G	p.H90R/p.MT-ATP6/TM	73.8	0	0.66	0	He	4
m.9972A > C	p.I256L/p.MT-CO3/IM	112.3	0.97	25.85	1	He	1
m.10543A > G	p.H25R/p.MT-ND4L/TM	150.5	2.57	0.66	0	He	4
m.10591 T > G	p.F41C/p.MT-ND4L/TM	134.9	0.02	1.08	0	He	3
m.12848C > T	p.A171V/p.MT-ND5/TM	164.3	0.16	8.93	0	He	3
m.13051G > A	p.G239S/p.MT-ND5/TM	163.9	0.02	23.65	0	Ho	4
m.13511A > T	p.K392M/p.MT-ND5/TM	98.9	0,02	3.24	0	He	4
m.13849A > C	p.N505H/p.MT-ND5/TM	61.8	0.27	5.24	0	Ho	2
m.14430A > G	p.W82R/p.MT-ND6/M	99.2	0.19	14.99	0	Ho	3
m.14498 T > C	p.Y59C/p.MT-ND6/TM	143.9	0.04	2.97	0	He	3
m.15243G > A	p.G166E/p.MT-CYB/IM	115.6	0	11.92	0	He	4
	Mean	118.8	0.48	10.25			
	Mean of neutral variants from validation dataset	80.1	7.90	12.70			

rCRS Mut, AA subs, PP, Dom, F1, F2, F3, Freq, Ho/He and DamPre code for position of the mutation according to the revised Cambridge Reference Sequence, amino acid substitution, polypeptide, domain, Feature 1–3 scores, frequency, Homoplasmy/Heteroplasmy, and number of predictors that consider damaging this amino acid substitution, respectively

### Prediction for all possible missense variants in the 13 human mtDNA-encoded polypeptides

We also provide the pathogenicity scores for all 24,201 possible amino acid changes in the 13 human mtDNA-encoding polypeptides. The revised Cambridge reference sequence (rCRS, NC_012920.1) was used to define the reference amino acid sequence of each gene. The results show that 15,049 (62.2%) potential missense substitutions due to single point mutations would be damaging (Additional file 14: Table S10).

Damaging predictions of Mitoclass.1 do not accumulate in particular genes. Despite 77.2% of confirmed pathologic mutations from mdmv.1 affecting four genes (*MT-ND1*, *ND5*, *ND6*, *ATP6*), only 30.2% of potential pathologic mutations affect these genes (close to the expected 34.9% considering the number of amino acids of these polypeptides) (Table 5A). This biased result of mdmv.1 dataset could indicate that phenotypes associated to mutations in some mtDNA genes (*MT-ND2-4*, *MT-CYB*, *MT-CO1-3*, and *MT-ATP8*) are not easily recognizable as mitochondriopathies and mtDNA pathologic mutations are not looked for.

**Table 5:** Percentage of confirmed and predicted pathologic mutations per polypeptide/complex (A) or domain (B)

A							
Complex	Polypeptide	AA	%	MUT	%	MUT	%
				Confirmed		Predicted	
CI		2214	55.8	36	63.2	7190	47.8
	p.MT-ND1	318	8.4	15	26.3	1300	8.6
	p.MT-ND2	347	9.2	1	1.8	1032	6.9
	p.MT-ND3	115	3.0	2	3.5	420	2.8
	p.MT-ND4	459	12.1	3	5.3	1689	11.2
	p.MT-ND4L	98	2.6	1	1.8	377	2.5
	p.MT-ND5	603	15.9	7	12.3	2008	13.3
	p.MT-ND6	174	4.6	7	12.3	364	2.4
CIII		380	10.0	2	3.5	1721	11.4
	p.MT-CYB	380	10.0	2	3.5	1721	11.4
CIV		1001	26.4	4	7.0	5146	34.2
	p.MT-CO1	513	13.5	1	1.8	2803	18.6
	p.MT-CO2	227	6.0	2	3.5	1021	6.8
	p.MT-CO3	261	6.9	1	1.8	1322	8.8
CV		294	7.8	15	26.3	992	6.6
	p.MT-ATP6	226	6.0	15	26.3	876	5.8
	p.MT-ATP8	68	1.8	0	0	116	0.8
B							
Domain		AA	%	MUT	%	MUT	%
				Confirmed		Predicted	
		3889	100	57	100	15049	100
IM		747	19.2	4	7.0	2883	19.1
TM		2376	61.1	42	73.7	9294	61.8
M		766	19.7	11	19.3	2872	19.1

Complex, polypeptide, AA, %, MUT, %, confirmed, predicted, IM, TM and M refer to OXPHOS complexes, mtDNA-encoded polypeptides, number of amino acids and its percentage in a particular polypeptide or domain, number of damaging mutations and its percentage in a particular polypeptide or domain, confirmed damaging mutations, predicted damaging mutations by Mitoclass.1, intermembrane, transmembrane and matrix domains, respectively

When analyzing presence of damaging mutations in each domain, the confirmed damaging mutations of mdmv.1 tend to be overrepresented in the transmembrane domain. However, the distribution of total damaging predictions does not accumulate in any particular domain. Despite 73.7% of confirmed mutations from mdmv.1 affecting transmembrane domain, only 61.8% of predicted variants affect this domain (similar to the expected 61.1% according to the number of amino acids in the domain). Predicted damaging mutations from matrix and intermembrane domains are also similar to the expected number (Table 5B). The explanation can be similar to the previous one. Maybe, mutations out of the transmembrane domain are not easily recognizable as mitochondriopathies and, therefore, mtDNA is not checked.

## Conclusions

We have developed a SVM classifier, Mitoclass.1, to predict pathogenicity of human mtDNA missense variants. This tool is a good screening classifier to select candidate damaging mtDNA missense mutations from patients suffering mitochondrial disorders, but taking into account that the real phenotypic effect of these variants must be always confirmed by functional analysis. We have trained and validated our model with a curated dataset of mtDNA amino acid substitutions instead of using benchmark datasets of nuclear variants. Because a well-curated dataset of mtDNA variants did not exist, we have established a set of pathogenicity criteria to develop the dataset, that we have called mdmv.1. The chosen discriminatory attributes are based on conservation and coevolution, but also introducing the novel idea of analyzing each polypeptide domain separately. The training of our predictor only with previously curated mtDNA variants as well as the selection of discriminatory features improves the performance when compared with other existing predictors. Finally, we have also provided predictive results with our classifier for all possible missense mutations of the thirteen polypeptides encoded by human mtDNA.

The number of mtDNA reference sequences from different species published in GenBank and the number of candidate mutations identified by sequencing is growing very fast. Because our discriminatory features are dependent on this information, and our predictor can be easily updated, Mitoclass.1 will be improved periodically by retraining with new data.

## Additional files

Additional file 1: Table S1. MtDNA missense variants.1 dataset (mdmv.1). Each mutation is described by its polypeptide RefSeq code (Code), gene (GENE), amino acid position within the polypeptide (AA position), wild type amino acid (WT AA), mutant amino acid (M AA), domain (Domain) and phenotype of the mutation (Phenotype). Numerical scores and predicted phenotypes for the three tested predictors (Provean, Mutpred and PolyPhen-2) are also included. (XLS 608 kb)
Additional file 2: Table S2. Domains (segments of amino acids) of mtDNA-encoded polypeptides. IM, TM and M code for intermembrane, transmembrane and matrix domains, respectively. (DOC 38 kb)
Additional file 3: Table S3. Ortholog proteins with known crystal structure. For each human polypeptide, the organism with known crystal structure and the PDB code and polypeptide chain is included. (DOC 36 kb)
Additional file 4: Table S4. Matrix of relative frequencies of each amino acid for each domain. Scores obtained from the multiple alignments of Eukaryota orthologs of the thirteen polypeptides encoded by human mtDNA. Each sub-table contains the scores for a single domain (intermembrane-IM, transmembrane-TM and matrix-M). (XLS 49 kb)
Additional file 5: Table S5. Training set before oversampling with SMOTE and validation set (each one in a different sheet of the file). Each mutation is described by its gene (GENE), amino acid position within the polypeptide (AA position), wild type amino acid (WT AA), mutant amino acid (M AA), domain (Domain) and phenotype of the mutation (Phenotype). (XLS 316 kb)
Additional file 6: Table S6. Training set after oversampling with SMOTE. 1666 mutations are synthetic samples. Each mutation is described by its phenotype of the mutation (Phenotype), numerical scores for the three features (Feature_1, Feature_2, Feature_3), gene (GENE), amino acid position within the polypeptide (AA position), wild type amino acid (WT AA), mutant amino acid (M AA) and domain (Domain). (XLS 427 kb)
Additional file 7: Figure S1. Representation of synthetic (SMOTE instances, red) and original (real instances, blue) dataset in the feature space. (DOC 62 kb)
Additional file 8: Table S7. Conservation Index (CI) in Eukaryota. Each score is described by its gene (GENE), amino acid position within the polypeptide (AA position) and wild type amino acid (WT AA) as well as number of ortholog sequences used in the multiple sequence alignment (Sequences). CI is described as a percentage. The revised Cambridge reference sequence (rCRS, NC_012920.1) was used to define the reference amino acid sequence of each gene. (XLS 453 kb)
Additional file 9: Figure S2. Correlation between features. These 2D-scatter-plots show lack of correlations between the features. Neutral and pathological classes are indicated by blue and red color, respectively. (DOC 62 kb)
Additional file 10: Table S8. Prediction of validation dataset. Each mutation is described by its phenotype (Mutation type) and numerical values of attributes (feature 1, 2, and 3). The affected polypeptides and amino acid are indicated with the Gene, WT AA, M AA, AA position and Domain columns that refers to gene, wild type and mutant type amino acid, position in the polypeptide and domain. A column with conservation index values for each variant is also included (CI column). Performance of predictors (Mitoclass.1, Provean, Mutpred and PolyPhen-2) shows numerical scores and/or predicted phenotypes. (XLS 292 kb)
Additional file 11: Figure S3. Venn diagram for predictive results of 23 pathological-confirmed variants from the validation dataset. (DOC 92 kb)
Additional file 12: Table S9. Amino acid substitutions with no clear evidences of pathogenicity. Mutations are described by the polypeptide amino acid position (AA Position), wild type amino acid (WT AA), mutant amino acid (MUT AA) and affected gene (Gene). Predicted phenotype of Mitoclass.1 is included. The next four columns summarize the prediction result (P, positive/damaging; N, negative/neutral) for the four tested predictors. The last column (Count) indicates the number of predictors with the same damaging phenotype output. (XLS 31 kb)
Additional file 13: Figure S4. Venn diagram showing unique and common damaging predictions among different predictors for amino acids substitutions with no clear evidences of pathogenicity. (DOC 93 kb)
Additional file 14: Table S10. Prediction results for all possible missense variants in the 13 human mtDNA-encoded polypeptides. Each mutation is described by its position in the revised Cambridge reference sequence (rCRS, NC_012920.1), base change, gene, codon position, wild type amino acid (WT AA), mutant amino acid (M AA), polypeptide amino acid position (AA position) and domain. Feat 1 to 3 indicates the numerical values for the four analyzed attributes. Mitoclass.1 prediction for each mutation is showed as well as PolyPhen-2 prediction and score. (XLS 4726 kb)

## Abbreviations

CI: Conservation index
cMI: Cumulative mutual information
FN: False negative
FP: False positive
IM: Intermembrane
LHON: Leber hereditary optic neuropathy
M: Matrix
MI: Mutual information
mtDNA: Mitochondrial DNA
nDNA: Nuclear DNA
NUMT: Nuclear mitochondrial DNA segment
OXPHOS: Oxidative phosphorylation
rCRS: Revised Cambridge reference sequence
ROS: Reactive oxygen species
SVM: Support vector machine
TM: Transmembrane
TN: True negative
TP: True positive
