# Supplementary material for: Machine learning classifier for identification of damaging missense mutations exclusive to human mitochondrial DNA-encoded polypeptides
Source: BMC Bioinformatics. 2017 Mar 7;18:158. doi: 10.1186/s12859-017-1562-7 (PMC5341421; doi:10.1186/s12859-017-1562-7)
Supplement: Additional file 2: Table S2. — Domains (segments of amino acids) of mtDNA-encoded polypeptides. IM, TM and M code for intermembrane, transmembrane and matrix domains, respectively. (DOC 38 kb) [file 12859_2017_1562_MOESM2_ESM.doc]

| **Human**  **Polypeptides** | **N-terminal**  **Location** | **Domains: Polypeptide segments** |
| --- | --- | --- |
| **p.MT-ND1** | TM | IM: 84-102; 159-178; 179-192; 241-256; 314-318 TM: 1-27; 74-83; 103-122; 128-158; 219-240; 257-276; 283-313 M: 28-73; 123-127; 193-218; 277-282 |
| **p.MT-ND2** | IM | IM: 1-2; 44-48; 123; 170-174; 223-237; 300-313 TM: 3-21; 26-43; 49-80; 93-122; 124-144; 148-169; 175-190; 200-222; 238-273; 276-299; 314-332 M: 22-25; 81-92; 145-147; 191-199; 274-275; 333-347 |
| **p.MT-ND3** | TM | IM: 79-83 TM: 1-21; 53-78; 84-107 M: 22-52; 108-115 |
| **p.MT-ND4** | IM | IM: 1-7; 42-68; 112-114; 172-185; 247-252; 303-305; 388; 448-459 TM: 8-18; 23-41; 69-88; 96-111; 115-134; 142-171; 186-207; 228-246; 253-275; 282-302; 306-336; 355-387; 389-414; 428-447 M: 19-22; 89-95; 135-141; 208-227; 276-281; 337-354; 415-427 |
| **p.MT-ND4L** | IM | IM: 1; 53-54 TM: 2-21; 25-52; 55-82 M: 22-24; 83-98 |
| **p.MT-ND5** | IM | IM: 1-14; 64-83; 134-136; 191-192; 265-271; 320-321; 401-405; 463-494 TM: 15-33; 42-63; 84-107; 114-133; 137-156; 162-190; 193-240; 241-264; 272-291; 298-319; 322-350; 368-400; 406-431; 449-462; 495-521; 594-603 M: 34-41; 108-113; 157-161; 292-297; 351-367; 432-448; 522-593 |
| **p.MT-ND6** | IM | IM: 1-2; 47-48; 111-135 TM: 3-21; 25-46; 49-74; 91-110; 136-157 M: 22-24; 75-90; 158-174 |
| **p.MT-CYB** | M | IM: 53-75; 131-174; 245-286; 341-344;  TM: 33-52; 76-104; 110-130; 175-201; 222-244; 287-309; 322-340; 345-376;  M: 1-32; 105-109; 202-221; 310-321; 377-380 |
| **p.MT-CO1** | M | IM: 42-51; 118-141; 214-229; 285-298; 359-371; 436-448 TM: 12-41; 52-83; 95-117; 142-170; 183-213; 230-262; 270-284; 299-327; 336-358; 372-396; 407-435; 449-478 M: 1-11; 84-94; 171-182; 263-269; 328-335; 397-406; 479-513 |
| **p.MT-CO2** | IM | IM: 1-14; 89-227 TM: 15-46; 62-88 M: 47-61 |
| **p.MT-CO3** | M | IM: 36-40; 108-128; 184-191; 256-261 TM: 16-35; 41-65; 73-107; 129-153; 156-183; 192-224; 233-255 M: 1-15; 66-72; 154-155; 225-232 |
| **p.MT-ATP6** | IM | IM: 1-7; 71-85; 178-184 TM: 8-25; 52-70; 86-121; 151-177; 185-221 M: 26-51; 122-150; 222-226 |
| **p.MT-ATP8** | IM | IM: 1-7 TM: 8-24 M: 25-68 |

Additional Table 2. Domains (segments of amino acids) of mtDNA-encoded polypeptides.
