# Supplementary material for: Machine learning classifier for identification of damaging missense mutations exclusive to human mitochondrial DNA-encoded polypeptides
Source: BMC Bioinformatics. 2017 Mar 7;18:158. doi: 10.1186/s12859-017-1562-7 (PMC5341421; doi:10.1186/s12859-017-1562-7)
Supplement: Additional file 3: Table S3. — Ortholog proteins with known crystal structure. For each human polypeptide, the organism with known crystal structure and the PDB code and polypeptide chain is included. (DOC 36 kb) [file 12859_2017_1562_MOESM3_ESM.doc]

| **Human**  **Polypeptide** | **PDB code (chain)** | **Organism** |
| --- | --- | --- |
| **p.MT-ND1** | 4HEA (chain H) | *Thermus thermophilus* |
| **p.MT-ND2** | 4HEA (chain N) | *Thermus thermophilus* |
| **p.MT-ND3** | 4HEA (chain A) | *Thermus thermophilus* |
| **p.MT-ND4** | 4HEA (chain M) | *Thermus thermophilus* |
| **p.MT-ND4L** | 4HEA (chain K) | *Thermus thermophilus* |
| **p.MT-ND5** | 4HEA (chain L) | *Thermus thermophilus* |
| **p.MT-ND6** | 4HEA (chain J) | *Thermus Thermophilus* |
| **p.MT-CYB** | 1QCR (chain C) | *Bos taurus* |
| **p.MT-CO1** | 1OCC (chain A) | *Bos taurus* |
| **p.MT-CO2** | 1OCC (chain B) | *Bos taurus* |
| **p.MT-CO3** | 1OCC (chain C) | *Bos taurus* |
| **p.MT-ATP6** | 1C17 (chain M) | *Escherichia coli* |
| **p.MT-ATP8** | NA | NA |

NA, not available

Additional Table 3. Ortholog proteins with known crystal structure.
