# Supplementary material for: Machine learning classifier for identification of damaging missense mutations exclusive to human mitochondrial DNA-encoded polypeptides
Source: BMC Bioinformatics. 2017 Mar 7;18:158. doi: 10.1186/s12859-017-1562-7 (PMC5341421; doi:10.1186/s12859-017-1562-7)
Supplement: Additional file 7: Figure S1. — Representation of synthetic (SMOTE instances, red) and original (real instances, blue) dataset in the feature space. (DOC 62 kb) [file 12859_2017_1562_MOESM7_ESM.doc]

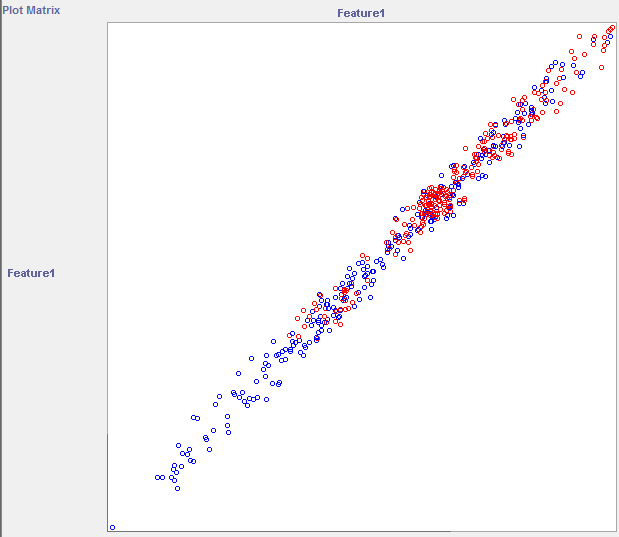


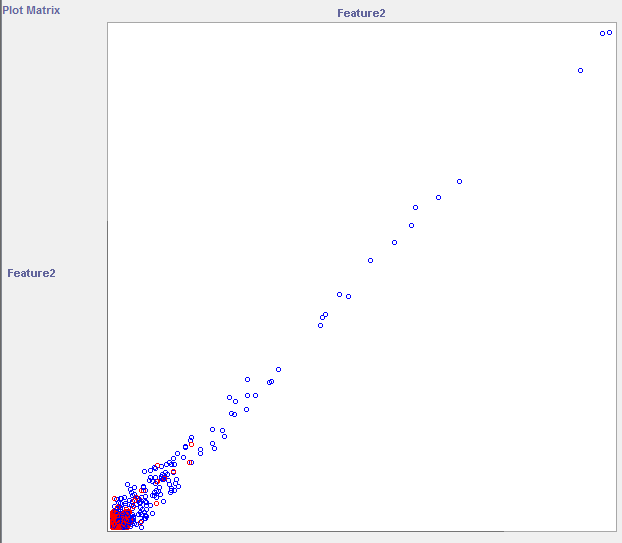


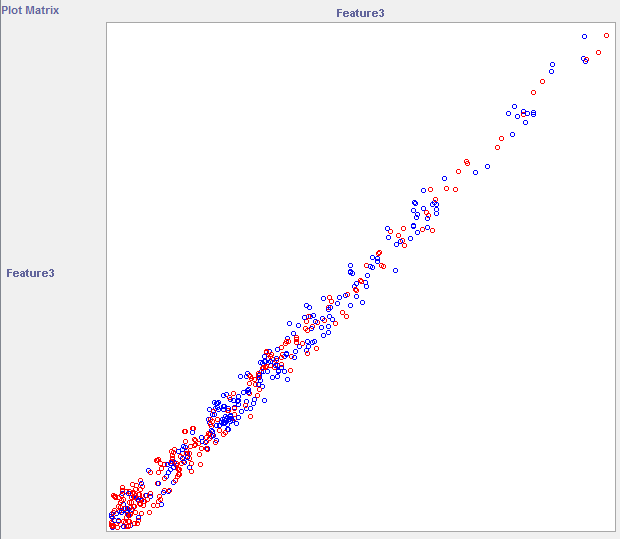


Additional Figure 1- Representation of synthetic (SMOTE instances, red) and original (real instances, blue) dataset in the feature space.
