# Supplementary figures and images for: Machine learning classifier for identification of damaging missense mutations exclusive to human mitochondrial DNA-encoded polypeptides
Source: BMC Bioinformatics. 2017 Mar 7;18:158. doi: 10.1186/s12859-017-1562-7 (PMC5341421; doi:10.1186/s12859-017-1562-7)

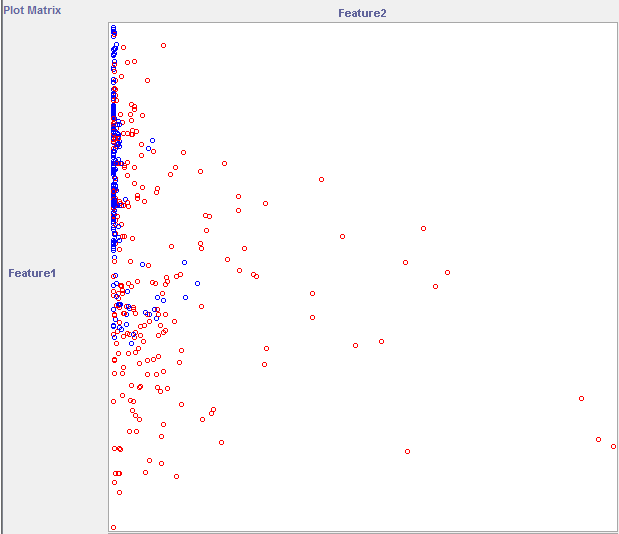


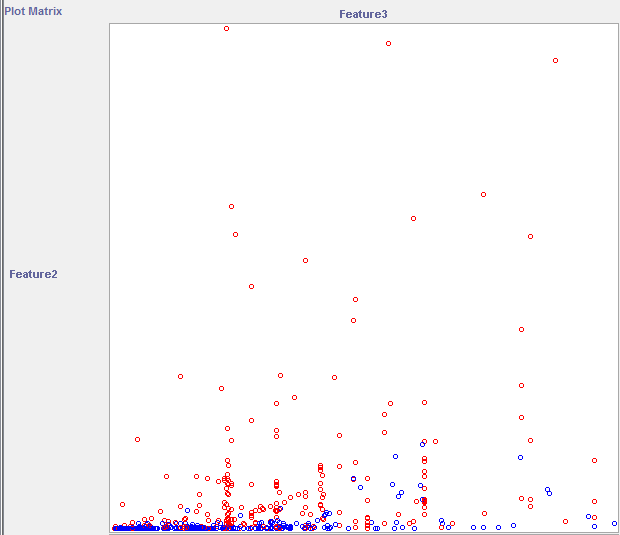


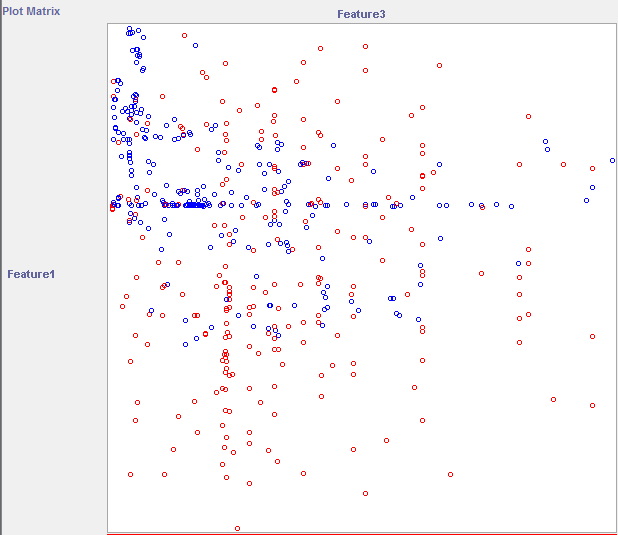


Additional Figure 2- Correlation between features.

Supplement: Additional file 9: Figure S2. — Correlation between features. These 2D-scatter-plots show lack of correlations between the features. Neutral and pathological classes are indicated by blue and red color, respectively. (DOC 62 kb) [file 12859_2017_1562_MOESM9_ESM.doc]
