# Supplementary material for: Machine learning classifier for identification of damaging missense mutations exclusive to human mitochondrial DNA-encoded polypeptides
Source: BMC Bioinformatics. 2017 Mar 7;18:158. doi: 10.1186/s12859-017-1562-7 (PMC5341421; doi:10.1186/s12859-017-1562-7)
Supplement: Additional file 11: Figure S3. — Venn diagram for predictive results of 23 pathological-confirmed variants from the validation dataset. (DOC 92 kb) [file 12859_2017_1562_MOESM11_ESM.doc]

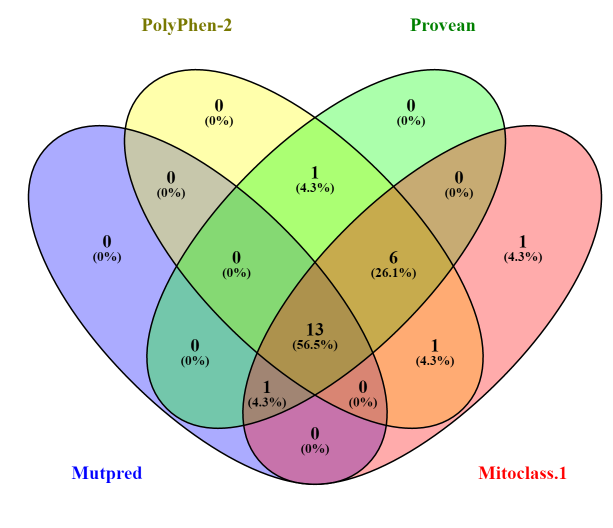


Additional Figure 3- Venn diagram for predictive results of 23 pathological-confirmed variants from the validation dataset.
