# Supplementary material for: Machine learning classifier for identification of damaging missense mutations exclusive to human mitochondrial DNA-encoded polypeptides
Source: BMC Bioinformatics. 2017 Mar 7;18:158. doi: 10.1186/s12859-017-1562-7 (PMC5341421; doi:10.1186/s12859-017-1562-7)
Supplement: Additional file 13: Figure S4. — Venn diagram showing unique and common damaging predictions among different predictors for amino acids substitutions with no clear evidences of pathogenicity. (DOC 93 kb) [file 12859_2017_1562_MOESM13_ESM.doc]

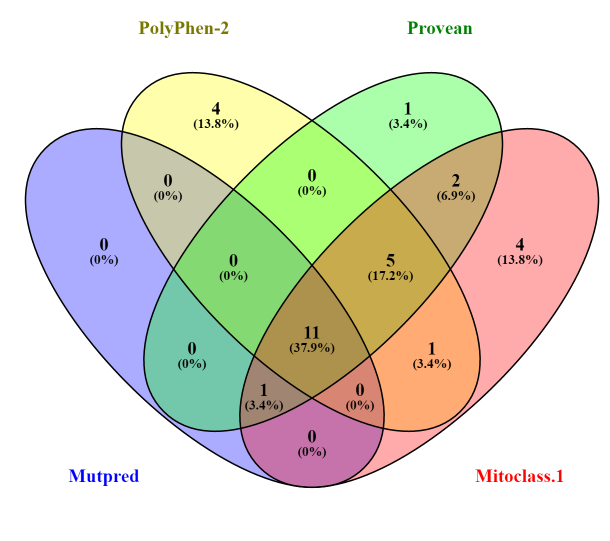


Additional Figure 4- Venn diagram showing unique and common damaging predictions among different predictors for amino acids substitutions with no clear evidences of pathogenicity.
